# Clinical features of patients with systemic sclerosis positive for anti-SS-A antibody: a cohort study of 156 patients

**DOI:** 10.1186/s13075-024-03325-6

**Published:** 2024-05-03

**Authors:** Tomoya Watanabe, Yasushi Ototake, Asami Akita, Mao Suzuki, Miwa Kanaoka, Jun Tamura, Yusuke Saigusa, Yukie Yamaguchi

**Affiliations:** 1 Department of Environmental Immuno-Dermatology, Yokohama City University Graduate School of Medicine, 3-9 Fukuura, Kanazawa- ku,Yokohama, 236-0004 Japan; 2 Department of Biostatistics, Yokohama City University Graduate School of Medicine, Yokohama, Japan

**Keywords:** Anti-SS-A antibody, Cohort study, Organ involvement, Systemic sclerosis

## Abstract

**Background:**

Anti-SS-A/Ro antibody (anti-SSA), the diagnostic marker of Sjögren’s syndrome (SS), is often detected in systemic sclerosis (SSc). Some patients are diagnosed with SSc/SS overlap syndromes, while there are anti-SSA-positive SSc cases without SS. In this study, we investigated the clinical characteristics of SSc with anti-SSA and clarified the clinical impact of this antibody in SSc.

**Methods:**

A retrospective chart review was conducted of 156 patients with SSc at Yokohama City University Hospital from 2018 to 2021. Clinical data, laboratory data, imaging, and autoantibody positivity status were collected and analysed to assess the association between these variables and anti-SSA using multivariable logistic regression analysis.

**Results:**

This cohort included 18 men and 138 women with SSc (median age, 69.0 years). Thirty-nine patients had diffuse cutaneous SSc (dcSSc) (25%), and 117 patients had limited cutaneous SSc (75%). Forty-four patients were anti-SSA-positive. Among them, 24 fulfilled the SS criteria. Multivariable logistic regression revealed that anti-SSA was statistically associated with interstitial lung disease (ILD; odds ratio [OR] = 2.67; 95% confidence interval [CI], 1.14–6.3; *P* = 0.024). Meanwhile, anti-SSA positivity tended to increase the development of digital ulcer (OR = 2.18; 95% CI, 0.99–4.82, *P* = 0.054). In the comparative analysis of the autoantibody single-positive and anti-SSA/SSc-specific autoantibody double-positive groups, the anti-SSA single-positive group showed a significantly increased risk of ILD (OR = 12.1; 95% CI, 2.13-140.57; *P* = 0.003). Furthermore, patients with SSc and anti-SSA indicated that anti-SSA-positive SSc without SS was strongly associated with dcSSc when compared to that in patients with SS (OR = 6.45; 95% CI, 1.23–32.60; *P* = 0.024).

**Conclusions:**

Anti-SSA positivity increases the risk of organ involvement, such as ILD, in patients with SSc. Additionally, the anti-SSA-positive SSc without SS population may have more severe skin fibrosis than others. Anti-SSA may be a potential marker of ILD and skin severity in SSc.

**Supplementary Information:**

The online version contains supplementary material available at 10.1186/s13075-024-03325-6.

## Background

Systemic sclerosis (SSc) is a multisystem connective tissue disease characterised by skin and internal organ fibrosis, microvascular dysfunction, and immune dysregulation [1]. The clinical phenotype of SSc is highly heterogeneous; thus, subgrouping the disease and predicting organ involvements are critical in clinical practice. The presence of distinctive circulating autoantibodies is another clinical feature of SSc. Specific autoantibodies are associated with unique cutaneous subtypes and risk profiles of internal organ involvements [2, 3]. For example, patients with anticentromere antibody (ACA) are often classified as having limited cutaneous SSc (lcSSc), whereas severe organ involvements, such as interstitial lung disease (ILD) and scleroderma renal crisis (SRC), occur rarely, except for pulmonary arterial hypertension (PAH). The majority of SSc patients positive for anti-topoisomerase I antibody (ATA) have diffuse cutaneous SSc (dcSSc), and ATA positivity is also associated with a high risk for ILD, cardiomyopathy, and digital ulcer (DU). Furthermore, rapidly progressive skin thickening and a higher risk of SRC have been reported in dcSSc patients with anti-RNA polymerase III antibody (RNAPIII) [2].

Anti-SS-A/Ro antibody (anti-SSA) is the diagnostic marker of Sjögren’s syndrome (SS) included in all classification criteria [4–6]. The SS-A antigen comprises two polypeptide components of 52 and 60 kDa. These autoantigens are referred to as “Ro52” and “Ro60” [7]. Anti-SSA is often detected in patients with other autoimmune diseases, such as systemic lupus erythematosus, rheumatoid arthritis (RA), and SSc. Some of these diseases are complicated by secondary SS diagnosed as overlap syndrome. Indeed, the rate of prevalence of SS in patients with SSc is 11–24% [8, 9]. In an analysis of the clinical phenotype of SSc/SS overlap syndrome, 83.6% of patients had lcSSc, and ILD was a less frequent complication [10]. The presence of anti-Ro antibodies was more likely to be related to this overlap syndrome, often with ACA positivity [11]. In contrast, some SSc patients are only positive for anti-SSA without symptoms of SS. Notably, a recent study revealed that the anti-Ro52 antibody may be a potential biomarker for lung fibrosis in mixed connective tissue disease [12]. Furthermore, the anti-PL7 and anti-Ro52 antibody combination is a predictive marker for rapidly progressive ILD in antisynthetase syndrome [13]. However, few studies have focused on patients with anti-SSA-positive SSc, and the clinical significance of this presentation is not fully understood.

In this study, we retrospectively investigated the clinical characteristics of patients with SSc positive for anti-SSA and clarified the clinical significance of this antibody in SSc.

## Methods

### Patients

Data were collected from retrospective chart review of Japanese patients diagnosed with SSc who received treatment at the Yokohama City University Hospital between January 2018 and July 2021. SSc patients fulfilled the 2013 American College of Rheumatology (ACR)/European League Against Rheumatism (EULAR) classification criteria for SSc [14]. SS patients met the 2016 ACR/EULAR classification criteria for primary Sjögren’s syndrome [6]. This study was approved by the Institutional Review Board of Yokohama City University (approval no.: F220100003).

### Clinical and biologic data

The collected data included age, sex, medical history, laboratory data, autoantibody positivity status, age at SSc onset defined as the date of the first non-Raynaud symptom attributable to SSc, SSc classification (lcSSc or dcSSc), the modified Rodnan total skin thickness score (mRSS) at the most severe stage, nailfold capillary abnormalities, and SSc-related organ involvement, such as ILD, DU, gastroesophageal reflux disease (GERD), PAH, Raynaud’s phenomenon, renal involvement, intestinal pseudo-obstruction, autoimmune hepatitis, and thyroiditis. Anti-SSA, ACA, ATA, and anti-U1RNP antibody (U1RNP) were detected using a chemiluminescent enzyme immunoassay. RNAPIII was detected using an enzyme-linked immunosorbent assay (SRL Inc. Tokyo, Japan). Disease duration was defined as from the diagnosis of SSc.

SSc-related organ involvement was defined as follows. ILD was defined as bilateral reticular opacity, ground-glass opacity, and/or a honeycomb appearance on chest high-resolution computed tomography. DU was defined as a defect in the epidermis and dermis in the distal interphalangeal surface of the proximal phalanx in an area measuring 2 mm or more in diameter [15]. PAH was defined as a mean pulmonary arterial pressure ≥ 25 mmHg and pulmonary vascular resistance of > 3 Wood units measured by right heart catheterisation after pulmonary hypertension caused by left-sided heart disease (pulmonary artery wedge pressure > 15 mmHg), advanced ILD (forced vital capacity (FVC) < 70% of predicted), or chronic thromboembolism had been excluded [16]. GERD was defined as symptoms and mucosal damage caused by abnormal reflux of stomach contents into the oesophagus, oral cavity, and lungs [17]. Intestinal pseudo-obstruction was defined as a radiological demonstration of wide-mouth colonic cystitis, impaired small intestinal motility, malabsorption, or small intestinal bacterial overgrowth requiring antibiotic treatment. Renal involvement was the onset of acute or subacute renal failure, often associated with accelerated hypertension and microangiopathic haemolytic anaemia.

### Statistical analysis

All continuous variables were expressed as mean ± standard deviation, and there were no missing data. Fisher’s exact, Mann-Whitney U tests, and Wilcoxon test were used for statistical analysis, as appropriate. We also performed a multivariable logistic regression analysis to investigate the effect of anti-SSA on organ involvement. ACA, ATA, U1RNP, and RNAPIII were used as explanatory variables in regression models. The Least Absolute Shrinkage and Selection Operator (LASSO) estimation method was employed to avoid overfitting and to select the predictive variables. We further examined the interactions between anti-SSA and other autoantibodies. Odds ratios (OR) were estimated using a logistic model in which the interaction terms of anti-SSA and other autoantibodies were added to the model obtained from the LASSO regression analysis. Firth’s method was used due to the small number of patients with double positivity for anti-SSA and other autoantibodies in the interaction model. All statistical analyses were conducted using the GraphPad Prism version 8.2 software (GraphPad Software, San Diego, CA, USA), JMP Pro 16.1.0 software (SAS Institute Inc., Cary, NC, USA), and R version 4.2.1 (R Foundation for Statistical Computing, Vienna, Austria).

## Results

### Baseline patient profiles

A total of 156 Japanese patients with SSc were included in this study. The median age was 69.0 (range, 14–97) years, and the mean disease duration was 13.3 ± 12.2 years. The cohort comprised 18 men (11.5%) and 138 women (88.5%). Thirty-nine patients (25.0%) were classified as having dcSSc, and 117 patients (75.0%) as having lcSSc. Seventy-two patients (46.1%) were positive for ACA, 27 (17.3%) for ATA, 14 (9.0%) for RNAPIII, and 14 (9.0%) for U1RNP. Those SSc-specific autoantibodies were not detected in 33 patients (21.1%). Forty-four patients (28.1%) tested positive for anti-SSA. Of those, 24 patients (15.4%) fulfilled the SS criteria and were classified as having secondary SS.

### Anti-SSA presence increased the risk of organ involvement

First, we examined the clinical characteristics of patients with anti-SSA-positive SSc. The patients were divided into anti-SSA-positive (*N* = 44) and anti-SSA-negative (*N* = 112). We summarized the clinical characteristics and results of the univariate analysis of the anti-SSA-positive and -negative groups in Table 1. Univariate analysis revealed that the anti-SSA-positive group had a significantly higher proportion of female patients (*P* = 0.024), longer disease duration (*P* = 0.001), and significantly higher incidence of DU (*P* = 0.011), ILD (*P* = 0.020), GERD (*P* = 0.012), and SS (*P* < 0.001) than the anti-SSA-negative group. Because organ involvement was strongly associated with SSc-specific and/or SSc-related autoantibodies, we performed a multivariable analysis of the recruited participants who developed ILD, GERD, and DU (Table 2). Multivariable analysis using LASSO revealed that anti-SSA was statistically associated with development of ILD (odds ratio [OR] = 2.67; 95% confidence interval [CI], 1.14–6.30; *P* = 0.024). Meanwhile, anti-SSA positivity was associated with an increased incidence of DU (OR = 2.18; 95% CI, 0.99–4.82, *P* = 0.054), although the differences were not statistically significant. As expected, ACA positivity was associated with a significantly decreased risk of developing ILD when compared to other autoantibodies (OR = 0.15; 95% CI, 0.06–0.36; *P* < 0.001), while U1RNP positivity was associated with a significantly increased incidence of GERD (OR = 5.38; 95% CI, 1.09–26.56; *P* = 0.0389). ATA positivity was associated with the risk of DU. These results suggest that anti-SSA presence in patients with SSc is a risk factor for organ involvement, particularly ILD.

**Table 1:** Comparison of clinical characteristics between SSc patients with and without anti-SS-A antibody

Variable	SSc total (*N* = 156)	Anti-SSA positive (*N* = 44)	Anti-SSA negative (*N* = 112)	*P*-value
**Sex**				
Men	18	1 (2.3%)	17 (15.2%)	0.024*
Women	138	43 (97.7%)	95 (84.8%)	
Age (years), median (IQR)	69.0 (58–77)	69.0 (60–79)	70.0 (57–77)	0.392
Disease duration (years), mean ± SD	13.3 ± 12.2	18.0 ± 14.2	11.4 ± 10.8	0.001*
**Type of SSc**				
lcSSc	117	31 (70.5%)	86 (76.8%)	0.419
DcSSc	39	13 (29.5%)	26 (23.2%)	
**SSc-specific autoantibody**				
Anticentromere antibody	72	17	55	0.286
Anti-topoisomerase I antibody	27	10	17	0.346
Anti-RNA polymerases III antibody	14	1	13	0.115
Anti-U1RNP antibody	14	10	4	0.031
Negative	33	10	23	0.828
**Organ involvement**				
mRSS mean ± SD	7.5 ± 10.3	9.0 ± 11.4	6.9 ± 9.8	0.240
Nailfold capillary abnormalities	122	32 (72.7%)	90 (81.8%)	0.271
Raynaud’s phenomenon	137	41 (93.2%)	96 (86.5%)	0.282
Digital ulcer	46	20 (45.5%)	26 (23.3%)	0.011*
Interstitial lung disease	72	27 (61.4%)	45 (40.2%)	0.020*
Gastroesophageal reflux disease	73	28 (63.6%)	45 (40.2%)	0.012*
Pulmonary arterial hypertension	13	6 (13.6%)	7 (6.3%)	0.194
Intestinal pseudo-obstruction	12	5 (11.4%)	7 (6.3%)	0.320
Renal involvement	2	0 (0.0%)	2 (1.8%)	1.000
Autoimmune hepatitis	21	6 (13.6%)	15 (13.4%)	1.000
Thyroiditis	6	3 (6.8%)	3 (2.7%)	0.351
SS complication	33	24 (54.5%)	9 (8.0%)	< 0.001*

IQR: interquartile range, SD: standard deviation

lcSSc: limited cutaneous systemic sclerosis, dcSSc: diffuse cutaneous systemic sclerosis

SS: Sjögren’s syndrome, mRSS: modified Rodnan total skin thickness score

**Table 2:** Multivariable logistic regression for the risk of organ involvement

Variable	ILD			GERD			DU		
	OR	95% CI	*P* value	OR	95% CI	*P* value	OR	95% CI	*P* value
**Anti-SSA**	2.67	1.14, 6.30	0.024*	1.73	0.79, 3.81	0.17	2.18	0.99, 4.82	0.054
ACA	0.15	0.06, 0.36	< 0.001*	NA			NA		
ATA	3.00	0.93, 9.67	0.065	NA			3.47	1.45, 8.55	0.005*
**RNAP III**	3.19	0.75, 13.53	0.116	NA			0.15	0.00, 1.12	0.084
U1RNP	NA			5.38	1.09. 26.56	0.039*	NA		
Age	NA			NA			0.98	0.95, 1.00	0.067
Sex	NA			NA			NA		
Disease Duration	NA			1.03	1.00, 1.06	0.056	1.03	1.00, 1.17	0.040*

ACA: anticentromere antibody, ATA: anti-topoisomerase I antibody, RNAP III: anti-RNA polymerase III antibody

U1RNP: anti-U1RNP antibody, ILD: interstitial lung disease, GERD: gastroesophageal reflux disease, DU: digital ulcer

OR: odds ratio, CI: confidence interval, NA: not applicable

### Comparison of the risk of organ involvement between anti-SSA/SSc-specific autoantibody double-positive patients and SSc-specific autoantibody single-positive patients

To examine whether anti-SSA positivity affects the clinical features of SSc-specific autoantibodies, we summarized the clinical characteristics and results of univariate analysis of the anti-SSA/SSc-specific autoantibody double-positive and SSc-specific autoantibody single-positive groups (Supplementary Table 1). Notably, the percentage of ILD cases in the anti-SSA/U1RNP double-positive group were significantly higher compared to that in the U1RNP single-positive group (*P* = 0.033). Moreover, the anti-SSA single-positive group exhibited a significantly higher percentage of ILD cases compared to the anti-SSA/SSc-specific autoantibody double-negative group (*P* = 0.021). The anti-SSA/ACA double-positive group showed a higher frequency of DU cases than the ACA single-positive group (*P* = 0.013). The anti-SSA/ATA double-positive group had a higher proportion of GERD and PAH cases than the ATA single-positive group (*P* = 0.046, 0.041, respectively). Regarding mRSS, it should be noted that there was a trend towards higher mRSS in the anti-SSA single-positive SSc and the ATA/anti-SSA double-positive and RNAPIII single-positive groups. To further explore the clinical characteristics of anti-SSA positive group, we compared anti-SSA/SSc-specific antibody double positive group to each SSc-specific antibody single positive group (Supplementary Table 2). The percentage of males in the anti-SSA/SSc-specific antibody double positive group was significantly lower compared to ATA and RNAPIII single-positive groups (*P* = 0.018, 0.007, respectively). Additionally, anti-SSA/SSc-specific antibody double positive group had lower frequency of dcSSc cases compared to RNAPIII single-positive group (*P* = 0.020), while it was significantly higher in ACA single-positive group (*P* = 0.006). Furthermore, anti-SSA/SSc-specific antibody double positive group had significantly higher frequency of DU cases compared to ACA and RNAPIII single-positive groups (*P* = 0.008, 0.002, respectively). Logistic regression analysis was further performed to identify the influence of the specific antibodies on organ involvement (Supplementary Table 3). In the single-positive groups, anti-SSA, ATA, and RNAPIII positivity significantly increased the risk of ILD development, whereas ACA positivity decreased this risk. The risk of DU development was also increased with ATA positivity. We also compared the risk of organ involvements for each SSc-specific autoantibody in the presence or absence of anti-SSA to determine the effect of anti-SSA (Supplementary Table 4). Interestingly, the risk of ILD development in the anti-SSA/U1RNP double-positive group was significantly increased compared to that in the U1RNP single-positive group (OR = 20.93; 95% CI, 1.32-333.19; *P* = 0.031). The risk of DU development was also increased with ACA positivity by the co-presence of anti-SSA (OR = 3.80; 95% CI, 1.19–12.12; *P* = 0.023). These results suggest that single-positive anti-SSA was strongly associated with ILD and that the combination of anti-SSA and other SSc-specific antibodies resulted in different clinical phenotypes.

**Table 3:** Comparison of clinical characteristics between anti-SSA-positive SSc patients with and without SS

Variable	SSc with SS (*N* = 24)	SSc without SS (*N* = 20)	*P*-value
**Sex**			
Men	0 (0.0%)	1 (5.0%)	0.455
Women	24 (100%)	19 (95.0%)	
Age (years), median (IQR)	69.5 (59–81)	68.5 (62–75)	0.450
Disease duration (years), mean ± SD	21.6 ± 14.3	13.7 ± 13.4	0.035*
**Type of SSc**			
lcSSc	21 (87.5%)	10 (50.0%)	0.009*
dcSSc	3 (12.5%)	10 (50.0%)	
**SSc-specific autoantibody**			
Anticentromere antibody	10	7	0.760
Anti-topoisomerase I antibody	4	6	0.472
Anti-RNA polymerases III antibody	1	0	1.000
Anti-U1RNP antibody	5	4	0.734
Negative	5	5	
Anti-SS-A/Ro antibody titer (U/mL), mean ± SD	1357 ± 2813	548 ± 1043	0.735
**Organ involvement**			
mRSS	5.0 ± 7.3	14.2 ± 13.5	0.053
Nailfold capillary abnormalities	15 (62.5%)	17 (85.0%)	0.173
Raynaud’s phenomenon	23 (95.8%)	18 (90.0%)	0.583
Digital ulcer	12 (50.0%)	8 (40.0%)	0.556
Interstitial lung disease	13 (54.2)	14 (70.0)	0.356
Gastroesophageal reflux disease	16(66.7%)	12 (60.0%)	0.757
Pulmonary arterial hypertension	4 (16.7%)	2 (10.0%)	0.673
Intestinal pseudo-obstruction	2 (8.3%)	3 (15.0%)	0.646
Renal involvement	0 (0.0%)	0 (0.0%)	1.000
Autoimmune hepatitis	4 (16.7%)	2 (10.0%)	0.673
Thyroiditis	1 (4.2%)	2 (10.0%)	0.582

IQR: interquartile range, SD: standard deviation

lcSSc: limited cutaneous systemic sclerosis, dcSSc: diffuse cutaneous systemic sclerosis

SS: Sjögren’s syndrome, mRSS: modified Rodnan skin score

### Anti-SSA-positive SSc with SS vs. anti-SSA-positive SSc without SS

We further investigated whether the presence or absence of SS affected the clinical characteristics of patients with anti-SSA-positive SSc (Table 3). All but one patient with anti-SSA-positive SSc were female. The proportion of dcSSc cases among SSc patients without SS was significantly higher than that among patients with SSc and SS (*P* = 0.009). Likewise, the mRSS in SSc patients without SS tended to be higher than that in SSc patients with SS (*P* = 0.053). No statistical significance was found in the proportion of other organ involvements. Notably, multivariable regression analysis showed that dcSSc was significantly associated with SSc without SS (OR = 6.45; 95% CI, 1.23–32.60; *P* = 0.024) (Table 4).

**Table 4:** Logistic regression analysis for the risk of anti-SSA-positive SSc patients without SS

Variable	OR	95% CI	*P*-value
Type of SSc (dcSSc)	6.45	1.28, 32.60	0.024*
Disease duration	0.98	0.93, 1.03	0.380
Nailfold capillary abnormalities	3.33	0.63, 17.53	0.157

OR: odds ratio

CI: confidence interval

dcSSc: diffuse cutaneous systemic sclerosis

## Discussion

This study examined the clinical significance of anti-SSA in a single-institutional cohort of patients with SSc. Statistical analyses revealed that the presence of anti-SSA was strongly associated with ILD complications in patients with SSc. Interestingly, the specific combination of anti-SSA and SSc-specific autoantibody increased the risk of organ involvement, such as ILD and DU, compared to that in patients with SSc-specific autoantibody alone. Notably, SSc patients positive for anti-SSA alone, without SSc-specific autoantibodies, exhibited a significantly higher risk of ILD and a tendency of higher mRSS. We further found that anti-SSA-positive SSc without SS was more strongly associated with dcSSc than anti-SSA-positive SSc with SS. Previous reports have suggested that anti-SSA positivity is generally more common in SSc/SS overlap syndrome with ACA positivity, lcSSc, and less complicated ILD [9, 10]. Our observations may provide a new and more detailed debate in this regard.

Several studies have indicated an association between anti-SSA and organ involvement in connective tissue diseases (CTD) including SSc [12, 13]. Meridor et al. reported that anti-SSA is an independent risk factor for a worse percentage of forced vital capacity (FVC) and a poorer prognosis in SSc patients [18]. In dermatomyositis, the presence of anti-SSA was associated with the development of a more severe ILD in patients with antisynthetase syndrome [19]. Furthermore, anti-SSA and anti-Jo-1 antibody double-positive was correlated with severe progressive ILD [20]. Meanwhile, several analyses focusing on the anti-Ro52 antibody instead of anti-SSA have been reported in recent years. Decker et al. showed that the anti-Ro52 antibody was independently associated with ILD in patients with CTD, irrespective of CTD type [21]. In Malaysia, the anti-Ro52 antibody was shown to be a promising biomarker for pulmonary involvement in patients with SSc, although the authors investigated a small cohort of patients with SSc [22]. In another study, anti-Ro52 antibody positivity was identified as an independent risk factor for the development of PAH in SSc patients [23]. Furthermore, a systematic review and meta-analysis revealed that the presence of anti-Ro52 antibody was associated with a higher frequency of ILD in patients with SSc (OR = 1.71; 95% CI: 1.04–2.83; *P* = 0.036) [24]. Notably, the disease severity of the lung measured using the Medsger Disease Severity Scale [25, 26] in patients with SSc who had anti-Ro52 antibody was worse than that in those without anti-Ro52 antibody [27]. The variations in these findings could be influenced by differences in the racial composition of included patients, severity assessment tools, and cohort size. While our study did not directly analyse the anti-Ro52 antibody in patients with SSc, we observed an increased risk of ILD associated with anti-SSA, consistent with previous findings. Therefore, it is plausible to extend our observations to the findings of the anti-Ro52 antibody.

Ro52 is a highly antigenic self-protein, and its reactivity may represent an epiphenomenon in tissues targeted by immune responses, especially the lungs [21, 28]. These strong immune responses and the subsequent tissue damage could contribute to developing ILD [21]. Ro52 is an interferon (IFN)-inducible E3 ligase that mediates the ubiquitination and proteasomal degradation of IFN regulatory transcription factors, resulting in the downregulation of pro-inflammatory cytokines [28–31]. Furthermore, the anti-Ro52 antibody inhibits E3 ligase activity and suppresses Ro52-regulated ubiquitination [32]. These mechanisms may lead to an increased production of pro-inflammatory cytokines and the induction of disease activity, including ILD. Ro60 is a component of small cytoplasmic ribonucleoprotein complexes. They can bind to misfolded, non-coding RNA-hY-RNA complexes and may be involved in their final degradation. In addition, Ro60 may be associated with cell survival after ultraviolet irradiation [33]. A recent study revealed that anti-Ro60 antibody positivity was more frequently noted in systemic lupus erythematosus and might be associated with more lupus anticoagulation and anti-cardiolipin antibodies [34]. However, the role of anti-Ro60 antibodies in the pathogenesis of SSc remains unclear.

A significant association with anti-Ro52 antibodies was also found for DU complications in patients with SSc in Turkey [35]. Our results similarly showed the presence of anti-SSA tended to be associated with DU complications, although the difference was not statistically significant. Moreover, analysis of the anti-SSA and each SSc-specific autoantibody combination indicated that the anti-SSA/ACA double-positive group increased the risk of developing DU compared to the ACA single-positive group. Similarly, the risk of ILD development in the anti-SSA/U1RNP double-positive group was significantly elevated compared to that in the U1RNP single-positive group. Thus, the co-presence of anti-SSA with SSc-specific autoantibodies may have a distinct effect on the clinical phenotype compared to SSc-specific autoantibodies alone. As the frequency of organ involvement in patients with SSc depends on the type of SSc-specific autoantibody, it is critical to analyse the risk of anti-SSA positivity for each type of SSc-specific autoantibody.

Furthermore, our analysis revealed that anti-SSA-positive SSc without SS is strongly associated with the proportion of dcSSc cases. To our knowledge, this is the first study to investigate the association between the clinical phenotypes of SSc with and without SS. However, the role of anti-SSA in SSc without SS is poorly understood. Further clinical and basic research on the mechanisms underlying the clinical significance of anti-SSA presence in patients with SSc is required.

In this study, all patients were of Japanese descent (Asian). Racial differences play a significant role in SSc research. For instance, data from the European Scleroderma Trials and Research (EUSTAR) database have highlighted regional variations in SSc-related ILD concerning clinical presentation and prognosis [36]. Moreover, a meta-analysis including nine studies on SSc-related ILD across diverse races and regions indicated an association between the presence of the anti-Ro52 antibody and a high frequency of ILD [24]. These findings suggested that the presence of anti-SSA in patients with SSc may be associated with the development of ILD irrespective of racial backgrounds.

Our study had some limitations. First, the small number of patients with SSc included compared to the previously reported large cohort study. Second, we examined SSc-specific antibodies, the measurement of which is covered by Japanese insurance. However, certain SSc-specific antibodies, such as anti-U3 RNP and anti-Th/To antibodies, were not measured. Therefore, we could not accurately evaluate the association between anti-SSA and some SSc-specific autoantibodies. Third, this was a single-institution, retrospective study. Since university hospitals receive more patients with severe SSc than clinics and general hospitals, a selection bias cannot be ruled out. Fourth, the disease duration was significantly longer in anti-SSA-positive SSc with SS than in those without SSc. This finding suggests that it may take time to develop SS. Consequently, some cases of anti-SSA-positive SSc without SS may have been misclassified. In addition, we did not compare Sicca-inducing drugs between anti-SSA-positive SSc with and without SS groups. Finally, we did not examine the differences in clinical features of SSc among individuals with single-positive or double-positive anti-Ro52 and Ro60 antibodies. Thus, we could not elucidate the pathological significance of each protein in the context of SSc.

## Conclusions

Our findings demonstrate that ILD complications in patients with SSc were significantly increased in the presence of anti-SSA. Anti-SSA was also associated with different risks of organ involvement depending on the combination of SSc-specific autoantibodies. Additionally, the anti-SSA-positive SSc without SS population may have more severe skin fibrosis. These results suggest that anti-SSA may be a valuable biomarker of organ involvement and skin severity in patients with SSc. Hence, periodic screening for these involvements should be recommended in patients with SSc and anti-SS-A/Ro antibodies.

### Electronic supplementary material

Below is the link to the electronic supplementary material.

Supplementary Material 1

## Data Availability

All data generated or analysed during this study are included in this published article and available upon request from the corresponding author.

## Abbreviations

SS: Sjögren’s syndrome
SSc: Systemic sclerosis
dcSSc: diffuse cutaneous SSc
lcSSc: limited cutaneous SSc
ACA: Anticentromere antibody
ATA: Anti-topoisomerase I antibody
RNAPIII: Anti-RNA polymerase III antibody
U1RNP: anti-U1RNP antibody
ILD: Interstitial lung disease
SRC: Scleroderma renal crisis
PAH: Pulmonary arterial hypertension
DU: Digital ulcer
IPO: Intestinal pseudo-obstruction
GERD: Gastroesophageal reflux disease
mRSS: modified Rodnan total skin thickness score
RA: Rheumatoid arthritis
CTD: Connective tissue diseases
IFN: Interferon
ACR: American College of Rheumatology
EULAR: European League Against Rheumatism
LASSO: Least Absolute Shrinkage and Selection Operator
OR: Odds ratios
CI: Confidence interval
