# Supplementary material for: Clinical features of patients with systemic sclerosis positive for anti-SS-A antibody: a cohort study of 156 patients
Source: Arthritis Res Ther. 2024 May 3;26:93. doi: 10.1186/s13075-024-03325-6 (PMC11067241; doi:10.1186/s13075-024-03325-6)
Supplement: Supplementary file 1 — Supplementary Material 1 [file 13075_2024_3325_MOESM1_ESM.docx]

**Supplementary Table 1. Comparison of clinical characteristics between SSc-specific autoantibody with and without anti-SSA**

| Variable | ACA (+) | | | ATA (+) | | | RNAP III (+) | | | |
| --- | --- | --- | --- | --- | --- | --- | --- | --- | --- | --- |
|  | Anti-SSA (+) (N = 17) | Anti-SSA (-) (N = 55) | *P* value | Anti-SSA (+) (N = 10) | Anti-SSA (-) (N = 17) | *P* value | Anti-SSA (+) (N = 1) | Anti-SSA (-) (N = 13) | *P* value |  |
| Sex |  |  |  |  |  |  |  |  |  |  |
| Men | 0 | 2 | 1.000 | 1 | 4 | 0.621 | 0 | 4 | 1.000 |  |
| Women | 17 | 53 |  | 9 | 13 |  | 1 | 9 |  |  |
| Age (years), median (IQR) | 74.0  (67.0-79.0) | 72.0  (59.0-76.5) | 0.119 | 69.5  (64.3-82.0) | 61.0  (49.0-76.0) | 0.191 | 52 | 67.0  (62.0-73.0) |  |  |
| Type of SSc |  |  |  |  |  |  |  |  |  |  |
| lcSSc | 17 (100%) | 51 (92.7%) | 0.567 | 3 (30%) | 9 (52.9%) | 0.424 | 1 (100%) | 4 (30.8%) | 0.357 |  |
| dcSSc | 0 (0%) | 4 (7.3%) |  | 7 (70%) | 8 (47.1%) |  | 0 (0%) | 9 (69.2%) |  |  |
| Organ involvement |  |  |  |  |  |  |  |  |  |  |
| mRSS mean ± SD | 2.3± 3.0 | 3.9± 6.4 | 0.404 | 16.1 ± 12.3 | 10.2 ± 10.1 | 0.186 | 2 | 14.8 ± 12.4 | - |  |
| Nailfold capillary abnormalities | 12 (70.6%) | 49 (89.1%) | 0.116 | 8 (80.0%) | 11 (64.7%) | 0.666 | 1 (100%) | 7 (53.8%) | 1.000 |  |
| Raynaud's phenomenon | 16 (94.1%) | 51 (92.7%) | 1.000 | 9 (90.0%) | 16 (94.1%) | 1.000 | 1 (100%) | 6 (46.2%) | 0.468 |  |
| DU | 9 (52.9%) | 11 (20%) | 0.013* | 7 (70.0%) | 9 (52.9%) | 0.448 | 0 (0%) | 0 (0%) | 1.000 |  |
| ILD | 3 (17.6%) | 10 (18.2%) | 1.000 | 9 (90.0%) | 13 (76.5%) | 0.621 | 0 (0%) | 11 (84.6%) | 0.268 |  |
| GERD | 11 (64.7%) | 24 (43.6%) | 0.166 | 8 (80.0%) | 6 (35.3%) | 0.046* | 1 (100%) | 4 (30.8%) | 0.333 |  |
| PAH | 1 (5.9%) | 2 (3.6%) | 0.560 | 3 (30%) | 0 (0.0%) | 0.041* | 0 (0%) | 2 (15.4%) | 1.000 |  |
| IPO | 2 (11.8%) | 4 (7.3%) | 0.623 | 1 (10.0%) | 1 (5.9%) | 1.000 | 0 (0%) | 0 (0%) | 1.000 |  |
| Autoimmune hepatitis | 5 (29.4%) | 13 (23.6%) | 0.750 | 0 (0%) | 0 (0.0%) | 1.000 | 0 (0%) | 0 (0%) | 1.000 |  |
| Thyroiditis | 2 (11.8%) | 1 (1.8%) | 0.191 | 0 (0%) | 0 (0.0%) | 1.000 | 0 (0%) | 0 (0%) | 1.000 |  |
| SS complication | 10 (58.8%) | 7 (10.9%) | < 0.001* | 4 (40.0%) | 0 (0.0%) | 0.012* | 1 (100%) | 0 (0%) | 0.068 |  |

| Variable | U1RNP (+) | | | SSc-specific Ab (-) | | |
| --- | --- | --- | --- | --- | --- | --- |
|  | Anti-SSA (+) (N = 6) | Anti-SSA (-) (N = 4) | *P* value | Anti-SSA (+) (N = 10) | Anti-SSA (-) (N = 23) | *P* value |
| Sex |  |  |  |  |  |  |
| Men | 0 | 0 | 1.000 | 0 | 7 | 0.074 |
| Women | 6 | 4 |  | 10 | 16 |  |
| Age (years), median (IQR) | 65.0  (56.3-68.8) | 76.0  (64.3-78.5) | 0.762 | 66.5  (54.3-73.5) | 68.0  (51.0-77.5) | 0.931 |
| Type of SSc |  |  |  |  |  |  |
| lcSSc | 4 (66.7%) | 4 (100%) | 0.467 | 6 (60.0%) | 16 (69.6%) | 0.696 |
| dcSSc | 2 (33.3%) | 0 (0%) |  | 4 (40.0%) | 7 (30.4%) |  |
| Organ involvement |  |  |  |  |  |  |
| mRSS mean ± SD | 9.7 ± 7.1 | 1.0 ± 1.7 | 0.064 | 15.8 ± 15.4 | 8.5 ± 11.8 | 0.212 |
| Nailfold capillary abnormalities | 3 (50.0%) | 4 (100%) | 0.200 | 8 (80.0%) | 19 (82.6%) | 1.000 |
| Raynaud's phenomenon | 6 (100%) | 4 (100%) | 1.000 | 9 (90.0%) | 19 (82.6%) | 1.000 |
| DU | 1 (16.7%) | 1 (25.0%) | 1.000 | 3 (30.0%) | 5 (21.7%) | 0.673 |
| ILD | 6 (100%) | 1 (25.0%) | 0.033* | 9 (90.0%) | 10 (43.5%) | 0.021* |
| GERD | 5 (83.30%) | 3 (75.0%) | 1.000 | 3 (30.0%) | 8 (34.8%) | 1.000 |
| PAH | 0 (0%) | 0 (0%) | 1.000 | 2 (20.0%) | 3 (13.0%) | 0.627 |
| IPO | 0 (0%) | 0 (0%) | 1.000 | 2 (20.0%) | 2 (8.7%) | 0.567 |
| Autoimmune hepatitis | 1 (16.7%) | 0 (0%) | 1.000 | 0 (0%) | 3 (13.0%) | 0.539 |
| Thyroiditis | 0 (0%) | 0 (0%) | 1.000 | 1 (10.0%) | 1 (4.3%) | 0.521 |
| SS complication | 4 (66.7%) | 1 (25.0%) | 0.524 | 5 (50.0%) | 1 (4.3%) | 0.005* |

ACA: anticentromere antibody, ATA: anti-topoisomerase I antibody, RNAPIII: anti-RNA polymerase III antibody, U1RNP: anti-U1RNP antibody, Ab: antibody, ILD: interstitial lung disease, GERD: gastroesophageal reflux disease, DU: digital ulcer, mRSS: modified Rodnan skin score, PAH: Pulmonary arterial hypertension, IPO: Intestinal pseudo-obstruction, SS: Sjögren's syndrome

**Supplementary Table 2. Comparison of clinical characteristics between anti-SSA/SSc-specific autoantibody double-positive patients and SSc-specific autoantibody single-positive patients**

| Variable | Reference | Only SSc-specific Ab positive group | | | | | | | |
| --- | --- | --- | --- | --- | --- | --- | --- | --- | --- |
|  | Anti-SSA (+) (N = 44) | ACA (+) (N = 55) | *P* value | ATA (+) (N = 17) | *P* value | RNAP III (+) (N = 13) | *P* value | U1RNP (+) (N = 13) | *P* value |
| Sex |  |  |  |  |  |  |  |  |  |
| Men | 1 | 2 | 1.000 | 4 | 0.018* | 4 | 0.007* | 0 | 1.000 |
| Women | 43 | 53 |  | 13 |  | 9 |  | 4 |  |
| Age (years), median (IQR) | 69.0  (58.0-77.0) | 72.0  (59.0-76.5) | 0.884 | 61.0  (49.0-76.0) | 0.138 | 67.0  (62.0-73.0) | 0.367 | 76.0  (64.3-78.5) | 0.849 |
| Type of SSc |  |  |  |  |  |  |  |  |  |
| lcSSc | 31 (70.5%) | 51 (92.7%) | 0.006* | 9 (52.9%) | 0.236 | 4 (30.8%) | 0.020* | 4 (100%) | 0.562 |
| dcSSc | 13 (29.5%) | 4 (7.3%) |  | 8 (47.1%) |  | 9 (69.2%) |  | 0 (0%) |  |
| Organ involvement |  |  |  |  |  |  |  |  |  |
| mRSS mean ± SD | 9.0 ± 11.4 | 3.9 ± 6.4 | 0.025* | 10.2 ± 10.1 | 0.626 | 14.8 ± 12.4 | 0.104 | 1.0 ± 1.7 | 0.075 |
| Nailfold capillary abnormalities | 32 (72.7%) | 49 (89.1%) | 0.064 | 11 (64.7%) | 0.546 | 7 (53.8%) | 0.307 | 4 (100%) | 0.559 |
| Raynaud's phenomenon | 41 (93.2%) | 51 (92.7%) | 1.000 | 16 (94.1%) | 1.000 | 6 (46.2%) | < 0.001* | 4 (100%) | 1.000 |
| DU | 20 (45.5%) | 11 (20%) | 0.008* | 9 (52.9%) | 0.775 | 0 (0%) | 0.002* | 1 (25.0%) | 0.621 |
| ILD | 27 (61.4%) | 10 (18.2%) | < 0.001* | 13 (76.5%) | 0.370 | 11 (84.6%) | 0.182 | 1 (25.0%) | 0.294 |
| GERD | 28 (63.6%) | 24 (43.6%) | 0.068 | 6 (35.3%) | 0.082 | 4 (30.8%) | 0.055 | 3 (75.0%) | 1.000 |
| PAH | 6 (13.6%) | 2 (3.6%) | 0.133 | 0 (0.0%) | 0.172 | 2 (15.4%) | 1.000 | 0 (0%) | 1.000 |
| IPO | 5 (11.4%) | 4 (7.3%) | 0.505 | 1 (5.9%) | 1.000 | 0 (0%) | 0.578 | 0 (0%) | 1.000 |
| Autoimmune hepatitis | 6 (13.6%) | 13 (23.6%) | 0.304 | 0 (0.0%) | 0.172 | 0 (0%) | 0.319 | 0 (0%) | 1.000 |
| Thyroiditis | 3 (6.8%) | 1 (1.8%) | 0.320 | 0 (0.0%) | 0.553 | 0 (0%) | 1.000 | 0 (0%) | 1.000 |

ACA: anticentromere antibody, ATA: anti-topoisomerase I antibody, RNAPIII: anti-RNA polymerase III antibody, U1RNP: anti-U1RNP antibody, Ab: antibody, ILD: interstitial lung disease, GERD: gastroesophageal reflux disease, DU: digital ulcer, mRSS: modified Rodnan skin score, PAH: Pulmonary arterial hypertension, IPO: Intestinal pseudo-obstruction

**Supplementary Table 3.** **Multivariate analysis for the risk of organ involvement in combination with anti-SSA and SSs-specific autoantibodies**

|  | Variable | ILD | | | GERD | | | DU | | |
| --- | --- | --- | --- | --- | --- | --- | --- | --- | --- | --- |
|  |  | OR | 95% CI | *P* value | OR | 95% CI | *P* value | OR | 95% CI | *P* value |
| Single positive | Anti-SSA (+) | 12.1 | 2.13, 140.57 | 0.003* | 0.81 | 0.16, 3.15 | 0.778 | 0.93 | 0.17, 4.47 | 0.925 |
|  | ACA (+) | 0.30 | 0.10, 0.85 | 0.023* | 1.42 | 0.54, 3.94 | 0.486 | 0.87 | 0.28, 2.93 | 0.812 |
|  | ATA (+) | 3.86 | 1.06, 16.02 | 0.040* | 1.03 | 0.28, 3.70 | 0.963 | 3.76 | 1.03, 15.08 | 0.045* |
|  | RNAP III (+) | 5.91 | 1.35, 35.80 | 0.017* | 0.86 | 0.20, 3.45 | 0.837 | 0.12 | 0.00, 1.26 | 0.083 |
|  | U1RNP (+) | 0.55 | 0.05, 3.99 | 0.564 | 4.25 | 0.59, 49.51 | 0.154 | 1.44 | 0.12, 11.33 | 0.741 |
| Double  positive | Anti-SSA (+)/ACA (+) | 0.09 | 0.01, 0.74 | 0.024* | 2.15 | 0.35, 14.91 | 0.415 | 4.09 | 0.64, 28.38 | 0.138 |
|  | Anti-SSA (+)/ATA (+) | 0.17 | 0.01, 3.14 | 0.218 | 6.62 | 0.73, 74.41 | 0.094 | 1.92 | 0.22, 19.16 | 0.558 |
|  | Anti-SSA (+)/RNAP III (+) | 0.01 | 0.00, 0.24 | 0.007* | 7.85 | 0.25, 143.34 | 0.245 | 9.72 | 0.04, 259.63 | 0.347 |
|  | Anti-SSA (+)/U1RNP (+) | 1.73 | 0.09, 41.66 | 0.720 | 2.26 | 0.11, 49.85 | 0.585 | 1.42 | 0.11, 25.39 | 0.792 |

ACA: anticentromere antibody, ATA: anti-topoisomerase I antibody, RNAPIII: anti-RNA polymerase III antibody, U1RNP: anti-U1RNP antibody, ILD: interstitial lung disease, GERD: gastroesophageal reflux disease, DU: digital ulcer,

OR: odds ratio, CI: confidence interval

**Supplementary Table 4. Comparison of the risk of organ involvement between anti-SSA/SSc-specific autoantibody double-positive patients and SSc-specific autoantibody single-positive patients**

| Variable | Reference | ILD | | | GERD | | | DU | | | |
| --- | --- | --- | --- | --- | --- | --- | --- | --- | --- | --- | --- |
|  |  | OR | 95% CI | *P* value | OR | 95% CI | *P* value | OR | 95% CI | *P* value |  |
| Anti-SSA (+)/ACA (+) | Anti-SSA (-)/ACA (+) | 1.09 | 0.28, 4.29 | 0.903 | 1.74 | 0.56, 5.40 | 0.337 | 3.80 | 1.19, 12.12 | 0.023* |  |
| Anti-SSA (+)/ATA (+) | Anti-SSA (-)/ATA (+) | 2.06 | 0.28, 15.36 | 0.482 | 5.36 | 0.96, 29.86 | 0.055 | 5.36 | 0.37, 8.61 | 0.470 |  |
| Anti-SSA (+)/RNAP III (+) | Anti-SSA (-)/RNAP III (+) | 0.12 | 0.004, 3.93 | 0.234 | 6.36 | 0.21, 188.94 | 0.285 | 9.04 | 0.13, 644.90 | 0.312 |  |
| Anti-SSA (+)/U1RNP (+) | Anti-SSA (-)/U1RNP (+) | 20.93 | 1.31, 333.19 | 0.031* | 1.83 | 0.14, 24.30 | 0.646 | 1.32 | 0.13, 13.17 | 0.816 |  |

ACA: anticentromere antibody, ATA: anti-topoisomerase I antibody, RNAPIII: anti-RNA polymerase III antibody, U1RNP: anti-U1RNP antibody, ILD: interstitial lung disease, GERD: gastroesophageal reflux disease, DU: digital ulcer,

OR: odds ratio, CI: confidence interval
